# Genome Sequence of the Human Opportunistic Fungus *Arthrocladium fulminans* (CBS 136243)

**DOI:** 10.1534/g3.119.400831

**Published:** 2020-04-01

**Authors:** Leandro Ferreira Moreno, Nickolas Menezes da Silva, Vinicius Almir Weiss, Flavia de Fátima Costa, Juliana V. Bittencourt, Luciano Medina Macedo, Renata R. Gomes, Emanuel M. Souza, Vania Aparecida Vicente, Benjamin Stielow, Sybren de Hoog

**Affiliations:** * Westerdijk Fungal Biodiversity Institute, Utrecht, The Netherlands,; † Institute of Biodiversity and Ecosystem Dynamics, University of Amsterdam, Amsterdam, Netherlands,; ‡ Graduate Program in Bioprocess Engineering and Biotechnology, Departament of Bioprocess Engineering and Biotechnology, Federal University of Paraná, Curitiba, PR, Brazil,; §§ Departament of Bioinformatics,; ** Graduate Program in Microbiology, Parasitology and Pathology, Department of Basic Pathology, Federal University of Paraná State, Curitiba, PR, Brazil,; § Center of Expertise in Mycology Radboudumc / CWZ, Nijmegen, The Netherlands,; †† Technological Federal University of Paraná, Ponta Grossa, PR, Brazil,; ‡‡ Department of Biochemistry and Molecular Biology, Federal University of Paraná State, Curitiba, PR, Brazil,

**Keywords:** black yeast, *Chaetothyriales*, *Trichomeriaceae*, whole-genome sequencing

## Abstract

The black yeast-like fungus *Arthrocladium fulminans* is known from strains that cause severe and eventually fatal disseminated infections in immunocompromised patients. Given the dramatic outcome of this clinical case, it is essential to understand the virulence potential of this species. The fungus is a member of the family *Trichomeriaceae*, at some phylogenetic distance from the *Herpotrichiellaceae* where most infectious fungi in the order *Chaetothyriales* are located. Main ecological preferences among *Trichomeriaceae* include colonization of exposed inert surfaces. Currently, black yeasts genomes that are available in public databases cover members of the families *Herpotrichiellaceae* and *Cyphellophoraceae*. In the present report, we sequenced the genome of the first member and only clinical representative of the family *Trichomeriaceae*.

*Arthrocladium fulminans* is a member of the fungal order *Chaetothyriales* that covers black yeasts and relatives, known for their potential to cause severe and mutilating infections in immunocompromised as well as in healthy humans. The order comprises five families: *Chaetothyriaceae*, *Cyphellophoraceae*, *Epibryaceae*, *Herpotrichiellaceae*, and *Trichomeriaceae* (Réblová *et al.* 2013). In general, black yeast-like fungi are believed to possess limited competitive abilities toward adjacent microbes (de Hoog 1993), probably having disadvantages for colonializing saprobic environments due to more rapidly growing competitors. Therefore, black yeasts have been commonly isolated from extreme habitats where, where interactions between organisms is limited due to the environmental stress. A remarkably large number of these species have been reported from human infections (Hoog *et al.* 2019). For example, chromoblastomycosis that affects skin and subcutaneous tissue is caused by clusters of species in the family *Herpotrichiellaceae*, and cerebral phaeohyphomycosis is mainly caused *Cladophialophora bantaina*, *Fonsecaea monophora*, *Rhinocladiella mackenziei* and *Exophiala dermatitidis* in the same family (Li and de Hoog 2009; Queiroz-Telles *et al.* 2016). The few cases of human infection reported outside *Herpotrichiellaceae* concern superficial skin diseases by members of *Cyphellophoraceae* (Réblová *et al.* 2013).

The family *Trichomeriaceae* comprises mainly rock-inhabiting and epiphytic species (Chomnunti *et al.* 2012). Phylogenetic analyses have demonstrated that rock-inhabiting fungi often form early diverging groups within the order *Chaetothyriales*. The non-virulent *Trichomeriaceae* may be ancestral to the opportunists in *Herpotrichiellaceae* (Gueidan *et al.* 2011). In contrast to most genera of Trichomeriaceae having consistent ecology (Selbmann *et al.* 2017) *Arthrocladium* includes very rare species with divergent ecological preferences. For example, *Arthrocladium tropicale* and *A. tardum* were isolated from ant domatia in *Leonardoxa africana* (Nascimento *et al.* 2016) and *A. caudatum* from leaf litter of Acacia karroo (Papendorf 1969). *Arthrocladium fulminans*. The single strain known of the latter species caused a fatal disseminated infection in a patient with a GATA-2 disorder, a rare genetic immunodeficiency syndrome (Egenlauf *et al.* 2019). In addition, *A. fulminans* was reported causing septic arthritis and osteomyelitis in an immunocompetent patient (Diallo *et al.* 2017).

Currently, only genomes of two derived families of *Chaetothyriales*, *i.e.*, *Cyphellophoraceae* and *Herpotrichiellaceae* have been sequenced and included in comparative genomic analyses. In order to determine the genomic composition of a basal linage of *Chaetothyriales*, we sequenced the genome of *A. fulminans* and functionally annotated their predicted proteins. Comparative analysis was done identifying orthologous clusters shared with other 23 black yeast species. Information about the genome of *A. fulminans* and members of other families will help to elucidate the origin of opportunism in *Herpotrichiellaceae*.

## Materials and Methods

### Strain and sequencing

To extract the genomic DNA, the fungus *Arthrocladium fulminans* CBS 136243 was cultured in malt extract broth (MEB), with shaking at 150 r.p.m. at 25° for 7 days. DNA was extracted via a cetyltrimethylammonium bromide (CTAB)-based method involving phenol-isoamyl alcoho/isoamyl alcohol (Möller *et al.* 1992). For genome sequencing, library construction (180 bases-insert library) and genome sequencing (Illumina HiSeq platform) were performed at Eurofins Genomics (Ebersberg, Germany).

### Assembly, annotation and comparative analysis

The read quality was assessed by FASTQC v. 0.11.7 (http://www.bioinformatics.babraham.ac.uk/projects/fastqc) and low-quality sequences were removed by Trimmomatic (Bolger *et al.* 2014) and adaptors were removed by BBDuk from the BBMap package (https://sourceforge.net/projects/bbmap/) High quality reads were assembled using SPAdes genome assembler v3.10.0 (Bankevich *et al.* 2012). To find de-novo repeats, the contig swere screened using RepeatModeler v1.0.8. To identify additional copies of *de novo* repeats across the genome assembly, the library produced by RepeatModeler was used as input for RepeatMasker v4.0.7. Genes were predicted by Augustus (Stanke and Waack 2003) using a training model generated by Genemark-ES v4.30 (Lomsadze 2005). Functional annotations where performed with InterProScan v5.27-66.0 (Quevillon *et al.* 2005) and BLAST against UniProt SwissProt database. Carbohydrate-active enzymes (CAZymes) were classified using the dbCAN2 meta server (Yin *et al.* 2012). The mitochondrial genome annotation was performed with MITOS pipeline (Bernt *et al.* 2013).

Cytochromes P450 genes (CYPs) were annotated by identification of proteins carrying the PFAM domain PF00067 using the InterProScan v5.27-66.0 (Quevillon *et al.* 2005). Putative CYP450 genes were organized into families and subfamilies as recommended by the International P450 Nomenclature Committee (Nelson 2006). The Mating Type locus (MAT) of *A. fulminans* was characterized by homology to the *MAT1-1* and *MAT1-2* reference sequences previously described in related species of *Herpotrichiellaceae*. (Teixeira *et al.* 2017). A comparative analyses of melanin-associated genes was done based on the Teixeira *et al.* (2017) using BLAST with e-value of 1 ☓ 10-5. The results is showed as the supplementary material (Table S1).

Orthologous groups were clustered by comparing the protein sequences of *Arthrocladium fulminans* to those of 23 previously sequenced black yeasts (Teixeira *et al.* 2017) using OrthoMCL pipeline (Li 2003) with a Markov inflation index of 1.5 and a maximum e-value of 1 ☓ 10-5. The single-copy genes were extracted of the OrthoMCL output.

### Species tree based on orthologous clusters

Single-copy orthologous protein sequences obtained with OrthoMCL were aligned with MUSCLE (Edgar 2004) and poorly aligned regions were automatically removed using TRIMAL (Capella-Gutierrez *et al.* 2009) under the “-automated1” setting. The sequences were concatenated with FASCONCAT (Kück and Meusemann 2010) v. 1.0 and species trees were inferred by maximum likelihood RAxML (Stamatakis 2006) using PROTGAMMABLOSUM62 and 1000 bootstraps of branch support.

### Data availability

*Arthrocladium fulminans* genome and the Mitochondrial genome have been deposited in the National Centre for Biotechnology Information (NCBI) under the accession numbers GCA_003614865.1 and MN593345. Supplemental material available at figshare: https://doi.org/10.25387/g3.11897388.

## Results and Discussion

### Assembly, completeness and content of the A. fulminans CBS 136243 genome

**Figure 1:**
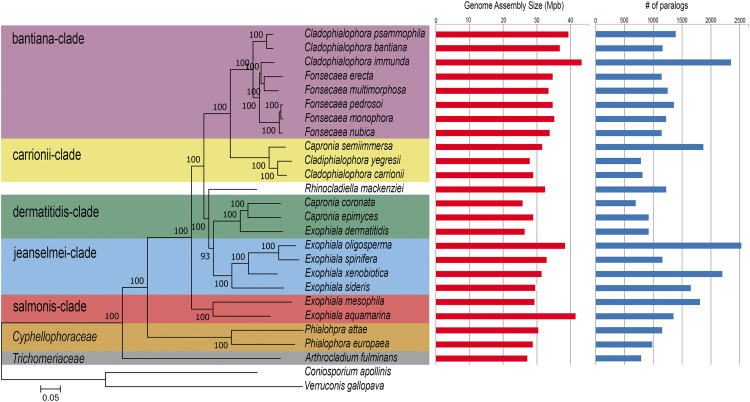
Genomic landscape of 23 black yeasts belonging to the order *Chaetothyriales* and the newly sequenced fungus *A. fulminans*. The species *Coniosporium apollinis* and *Verruconis gallopava* were used as outgroup for comparative genomic analyses.

The genome assembly of *Arthrocladium fulminans* CBS 136243 consists of 27 contigs comprising 27.22 Mb in size, read coverage of 54x and GC content of 51.82%, which is slightly above the average of 51.7% in black yeasts (Table 1). Comparable sizes are found in species belonging to the ‘dermatitidis-clade’ (de Hoog *et al.* 2011), a group of species with somewhat smaller genomes in *Herpotrichiellaceae*, where genomes vary between 25.81 Mb to 28.89 Mb in *Capronia coronata* and in *Capronia epimyces*, respectively (Figure 1). The repetitive portion of the genome comprises 426,983 bp (1.57%). Low repetitive contents are consistent across chaetothyrialean black yeasts, where repetitive elements are in the range of 0.03–2%.

**Table 1:** Genome Features

Type	Description	Value
Nuclear	Total sequence length	27,195,275
	Spanned gaps	2
	Number of scaffolds	27
	Scaffold N50	2,259,535
	Scaffold L50	6
	Number of contigs	34
	Contig N50	1,671,613
	Contig L50	7
Mitochondrion	Total sequence length	24,423
	Number of contigs	1

The completeness of the genome assembly of *A. fulminans*, was accessed with BUSCO (Simão *et al.* 2015). The genome assembly of A. fulminans contains 97.4%(295 of 303) complete BUSCO genes, being 293 (96.7%) complete and single-copy BUSCOs, 2 complete and duplicated BUSCOs (0.7%), 0,9% (5 of 295) fragmented genes and 3 missing genes.

### Genome annotation

The genome of *A. fulminans* CBS 136243 contains 315 genes coding for putative Carbohydrate-Active Enzymes (CAZymes), families of enzymes playing an essential role in the breakdown, biosynthesis and/or modification of a wide range of carbohydrates. This is the lowest reported number of CAZymes in *Chaetothyriales*, where they range between 339 genes in the opportunistic species *Exophiala dermatitidis* to 506 genes in the hydrocarbon-associated species *Exophiala xenobiotica*. This data suggests that the increased abundance and diversification of CAZymes in black yeasts is a recent evolutionary event in *Herpotrichiellaceae*. Similar to other species in the order *Chaetothyriales*, the Glycoside hydrolases (GH) superfamily is the most abundant class of CAZymes in *A. fulminans* with enlarged families being GH3 (*β*-glucosidase), GH31 (*α*-glucosidase), GH16 (xyloglucan: xyloglucosyltransferase), GH13-subfamily 40 (*α*-amylase) and GH18 (chitinase).

We identified 72 genes likely coding for CYPs in the genome of *A. fulminans*. This class of enzymes plays an essential role in primary and secondary metabolic pathways as well as in detoxification of xenobiotics. This repertoire of CYPs is comparable to that previously described in species of the ‘dermatitidis-clade’ (de Hoog *et al.* 2011). Overall, the CYPs were classified into 38 families and 49 subfamilies. Eleven CYPs could not be assigned to any family or subfamily and were considered unique among black yeasts. Among these genes, DZA80_5394 and DZA80_7723 seem to encode an isoform of the *CYP51A* and *CYP51B* highly conserved among *Eurotiomycetes* but absent in the order *Chaetothyriales*. The gene DZA80_8445 seems to be a P450nor, a unusual P450 reported in *Fusarium oxysporum* and responsible for reducing NO to N2O rather than catalyzing the monooxygenation reaction (Shiro *et al.* 1995). Comparative analyses of melanin-associated genes using data set previously reported by Teixeira *et al.* (2017) supported that yeasts have homologs for the production of melanin by the DHN pathway (Table S1).

The MAT locus of *A. fulminans* CBS 136243 is composed by a single copy of the *MAT1-2* gene (DZA80_6626), which contains the high mobility group box (HMG-box) domain (PF00505). This finding suggests a heterothallic (self-sterility) mating system. In this fungus, the flanking structure of this MAT locus resembles that of usually observed in *Eurotiomycetes* and includes, in its upstream region, the genes *APN2* (DZA80_6625), *COX13* (DZA80_6624) and *APC5* (DZA80_6623) (Table 2). A similar MAT structure containing the *COX13* gene has been only found in unrelated melanized fungi, such as *Verruconis gallopava* (*Venturiales*) and *Coniosporium apollinis* (incertae sedis); possibly *COX13* was lost in derived *Chaetothyriales* species. The gene *SLA2*, commonly found in the flanking region of the MAT locus in several *Eurotiomycetes* was found in a distinct genomic region.

**Table 2:** Genome annotation

Features	Value
Number of genes	8,534
Median gene length (bp)	1,838
Number of exons	19,553
Coding GC content	46.9%
Median exon length (bp)	722
Number of introns	11,022
Median intron length (bp)	140

### Comparative analysis

A substantial proportion of the predicted genes (4456) have homologs in other chaetothyrialean black yeast-like species. Two clusters of orthologs were specific to the ‘bantiana-clade’ and *A. fulminans*. These clusters correspond to a Carboxylesterase type B (IPR019819) and a Pectin lyase fold (IPR011050). Orthologs exclusively shared with the neurotropic species *Rhinocladiella mackenziei* include the amino acid transporter/polyamine (IPR002293), the Oxoglutarate/iron-dependent dioxygenase (IPR005123), metalloenzymes (IPR029068), Alcohol acetyltransferase/N-acetyltransferase (IPR010828), HNH nuclease (IPR003615), Short-chain dehydrogenase/reductase SDR (IPR002347) and the Aspartic peptidase (IPR021109) with multiple paralogs in *A. fulminans* and in *R.nbsp;mackenziei*. Seven orthologous clusters are shared between in *A. fulminans* and members of the family *Cyphellophoraceae*, *i.e.*, *Phialophora europaea* and *Phialophora attae*, including Carboxylesterase, type B (IPR002018) and Cytochrome P450 (IPR001128). Few orthologous clusters were found specifically among *A. fulminans* and the ‘salmonis-‘and ‘carrionii-clades’, however most of them without a known biological function. None specific orthologous clusters were found shared by *A. fulminans* and the ‘jeanselmei-clade’.

### Characteristics of the A. fulminans CBS 136243 mitochondrial genome

**Figure 2:**
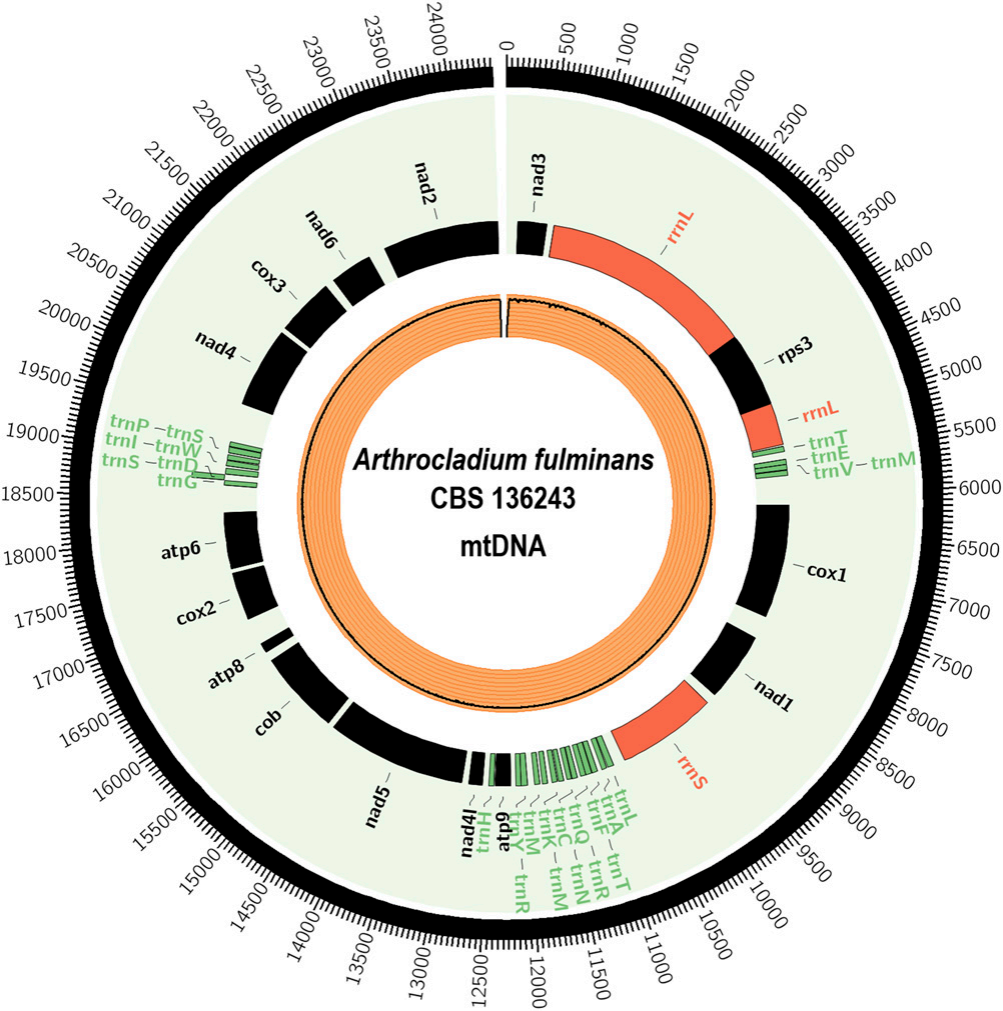
*Arthocladium Fulminans* CBS 136243 mtDNA. Rectangles represent annotated genes: in red rRNAs; in green tRNAs. Orange inner circle shows reads coverage.

The mitochondrial genome was assembled in a separate 24.5-kb contig with GC content of 27.3%. This sequence contains 25 tRNAs coding for all 20 standard amino acids, 15 polypeptide-encoding genes and two rRNA-encoding genes (Figure 2). The large variable region of the mitochondrial genome, between the genes rrnl and *nad2*, shows the presence of a *nad3* gene and the nuclear ribosomal protein S3 (*rps3*) which is anchored within the omega intron on the of the rrnl gene. The presence of mitochondrial *rps3* is reported in several other fungi and provides insight into its evolution (Korovesi *et al.* 2018), considered an ancient gene which seems to be evolved within the endosymbiotic model. According to the authors, the mt *rps3* proteins are descendants of archaeal and a-proteobacterial homologs, respectively. The nuclear ribosomal protein S3 (*rps3*) is implicated in the assembly of the ribosomal small subunit. Their C-terminal region is conserved in all Domains of life. Fungi and plants present a gene copy in their mitochondrial (mt) genomes which when *rps3* is found in fungal mt genomes, it is either a free-standing gene or an anchored gene within the omega intron.

## Conclusions

The whole genome sequence of the opportunistic, ancestral black yeast-like fungus *Arthrocladium fulminans* CBS 136243 was determined and compared with closely related species. Gene models were predicted and functionally annotated in order to identify gene families that likely led to the adaptation of extreme habitats by the black yeasts, such as CYPs and CAZymes. Our findings suggested that contrary to what was previously thought, gene family expansion took place later in the evolution of the black yeasts and the repertory of genes associated to resistance and nutrient uptake was reduced, but not absent, in basal lineages of black yeasts.
